# Disease Spectrum and Frequency of Illness in Pediatric Emergency: A Retrospective Analysis From Karachi, Pakistan

**DOI:** 10.31486/toj.18.0134

**Published:** 2019

**Authors:** Murtaza Gowa, Irfan Habib, Amber Tahir, Uzair Yaqoob, Sadaf Junejo

**Affiliations:** ^1^ Department of Pediatrics, National Institute of Child Health, Karachi, Pakistan; ^2^ Department of Pediatrics, Child Life Foundation, Karachi, Pakistan; ^3^ Sindh Medical College, Dow University of Health Sciences, Karachi, Pakistan

**Keywords:** *Emergencies*, *emergency medicine*, *emergency service–hospital*, *pediatric emergency medicine*, *pediatrics*

## Abstract

**Background:** The National Institute of Child Health (NICH) is the largest tertiary care pediatric hospital operating in Karachi, Pakistan. Its emergency department (ED) is always occupied. However, the spectrum of illness in patients presenting to this ED has not been investigated in depth to identify the most common presentations and to develop effective management for treating patients.

**Methods:** This retrospective study included all children visiting the pediatric ED of the NICH from January 2017 through December 2017. Newborns to children 14 years of age were included, for a total cohort of 188,803 patients. Sociodemographic data and clinical information were extracted from the medical record. Univariate analysis was performed to determine the frequency and percentage for all the variables.

**Results:** The cohort consisted of 9% (n=16,952) neonates (0 to 1 month) and 91% (n=171,351) older children (>1 month to 14 years). Among the neonates, 36.6% presented as triage level 1. Sepsis was diagnosed in 23.8% of neonates, low birth weight/preterm in 18.4%, and respiratory distress/pneumonia in 15.2%. In infants and older children, diagnoses related to the respiratory system (37.3%), gastrointestinal system (16.4%), and multisystem involvement (15.9%) were the most common. During the evening shifts, 38.1% of patients were seen, and on weekends, 51.6% of patients were seen. Sunday was the busiest day in the ED.

**Conclusion:** The tertiary care pediatric EDs in Pakistan have witnessed an increasing number of critical emergencies over time. Respiratory and gastrointestinal emergencies form the majority of the ED burden. A surge of patients is seen on the weekends and during the evening shifts. The spectrum of illnesses should be investigated via prospective, longitudinal studies in other pediatric EDs in Pakistan to understand the trends and to provide the foundation for developing nationwide recommendations for improving pediatric emergency care.

## INTRODUCTION

Pediatric emergency medicine is a critical aspect of pediatric healthcare, playing a pivotal role in improving childhood mortality from common illnesses.^1^ Emergency care focuses on urgent medical interventions in life-threating circumstances, so its quality is greatly determined by the decision-making skill of the healthcare provider and the necessary actions taken to prevent death or disability from time-critical illnesses.^2^

Every year, a growing number of critical childhood emergencies are reported from tertiary care hospitals worldwide. In 2010, 25 million children younger than 18 years (mean age of 7 years and principally males) were registered in United States emergency departments (EDs). Injuries, poisoning, and trauma were the most common presentations in children older than 5 years, while infections, including upper respiratory and fever of unknown origin, were most common in infants.^3^

The concept of emergency medicine is not new in developing countries; however, the implementation of evidence-based systems of care in pediatric and adult EDs is still new. For example, basic emergency services were the core healthcare priority in Mozambique after the country gained its independence in 1979, and basic emergency services are key elements of World Bank–funded health projects in Moldova and Romania.^4-6^

The lack of triage and immediate responsiveness to pediatric emergencies leads to potentially harmful delays in patient management.^7^ The World Health Organization (WHO) and the United Nations Children's Fund (UNICEF) have partnered to improve emergency medical care all over the world, especially in developing countries. Standardized protocols and replicable care models are being adapted from the developed world. In 2013, the WHO and UNICEF developed emergency triage, assessment, and treatment guidelines for the triage and care of critically ill children in developing countries.^8^ The guidelines, focused on low-resource settings where nonspecialists administer pediatric EDs, provide clinical guidance for managing severely ill infants and children.^8^ In a study conducted in Malawi by Robison et al, early hospital mortality in children was reduced from 47.6 to 37.9 per 1,000 admissions with improved training of hospital staff.^9^

Studies conducted in pediatric EDs in developing countries have shown predictable trends, with diagnoses of gastroenteritis, upper respiratory tract infections, pneumonia, and neurologic and neonatal emergencies accounting for most of the ED burden.^7,10^ Middle-income countries such as Pakistan have limited resources, extensive patient influx, and unavailability of real-time data about the spectrum of illnesses of the children presenting to the pediatric ED.^11^ To our knowledge, only one large-scale study^11^ has been conducted in Pakistan to assess the spectrum of illnesses in pediatric EDs, and 2 studies have investigated the severity of the diseases of children presenting to the EDs of pediatric tertiary care facilities.^12,13^ Habib and Khan reported in 2018 that 8% of children presenting to the pediatric ED were categorized as triage level 1 and required emergency life-saving interventions; most of them were neonates.^12^ Haque et al reported in 2015 that 1.7% of ED-registered children were admitted to the pediatric intensive care unit.^13^

An understanding of the disease spectrum in the pediatric EDs across Pakistan is still incomplete. Understanding the ED burden in terms of illnesses and injuries will allow practitioners to devise effective systems of service provision. The aim of this study was to determine the spectrum and frequency of illnesses in children presenting to the largest tertiary care pediatric ED in Pakistan.

## METHODS

The study was a retrospective analysis of all children managed in the ED of the National Institute of Child Health (NICH) in Karachi, Pakistan. Patients at all levels of triage, according to Emergency Severity Index,^14^ from January 1, 2017 through December 31, 2017 were included. The NICH is the largest public, pediatric, tertiary care referral hospital in Karachi. It is the national hub of the residency training program for general pediatrics and its subspecialties. This 500-bed hospital provides cost-free treatment to almost 1 million pediatric patients annually; 250,000 patients are managed in its ED annually, and almost 10,000 pediatric surgeries are performed at the NICH each year. The hospital serves patients from Karachi and the entire province of Sindh and most of the critically ill children from the province of Balochistan.^15^

All acutely ill children 0 to 14 years of age, whether self-, general practitioner–, or secondary care–referred, are seen in the NICH ED. The ED is a 55-bed unit with a well-functioning and efficient triage system, a resuscitation unit for critically ill children, a stepdown area for moderately ill children, a fast-track outpatient unit for stable children, a neonatal resuscitation unit, a procedure room, a minor emergency operating unit, and a pharmacy staffed with qualified pharmacists and cost-free emergency drugs.^15^ Imaging capabilities include x-rays and ultrasounds.

Registered nurses provide efficient nursing care. The ED is staffed with full-time senior registrars who are fellows of general pediatrics. On-call casualty medical officers (CMOs) are pediatric medicine residents trained in basic life support and pediatric advanced life support. According to the computerized registry in the ED, a total of 188,906 children were registered during the study period. Their ages ranged from the first day of life to 14 years. A single child may have had repeat visits during the 1-year period. The sociodemographic data and clinical information for the patients were extracted from the medical record, and the information was entered in Microsoft Excel v.2010. Univariate analysis was used to determine the frequencies and percentages for all variables.

## RESULTS

In 2017, 603 patients were registered and triaged but were not managed and left the NICH ED, resulting in a cohort of 188,303 patients who were registered, triaged, and managed: 16,952 (9.0%) neonates and 171,351 (91.0%) older children. The male to female ratio was 1.1:1. Sociodemographic and presentation variables are shown in Table 1. Most patients were seen during the evening shifts and on weekends. With a total of 71,654 children (38.1%) registered during evening shifts, the average number of daily patients registered in the evenings was 196. However, an analysis of the peak hours of patient burden showed that the greatest number of patients were seen between 11:00 am and 12:00 pm, yielding an average of 39 patients seen at this peak hour. The peak hours during the evening shifts were 6:00 pm to 8:00 pm, yielding a daily average of 32 patients being seen during the 6:00 pm to 7:00 pm time frame and another 35 patients, on average, being seen during the 7:00 pm to 8:00 pm time frame. The weekend to weekday ratio of patient influx was 1.3:1, with Sunday being the busiest day in the ED. A total of 28,460 patients (15.1%) were seen on Sundays, yielding an average of 547 patients seen every Sunday. Monday was the second busiest day with an average of 503 patients seen.

**Table 1.** Sociodemographic and Presentation Characteristics of the Patients Presenting to the National Institute of Child Health Emergency Department in 2017 (n=188,303)

	Number of
Variable	Patients (%)
Age	
0-1 month (neonates)	16,952 (9.0)
>1-12 months	58,471 (31.1)
>1-5 years	74,704 (39.7)
>5-10 years	30,766 (16.3)
>10 years	7,410 (3.9)
Sex	
Male	98,829 (52.5)
Female	89,342 (47.4)
Neonates with ambiguous genitalia	132 (0.1)
Residential area	
Resides in Karachi	94,741 (50.3)
Resides in Sindh	68,589 (36.4)
Resides in Balochistan	24,973 (13.3)
Mode of referral	
Self	48,654 (25.8)
Primary/secondary care institution in Karachi	78,025 (41.4)
Tertiary care institution in Karachi	10,469 (5.6)
Tertiary care institution in another city in Sindh	27,118 (14.4)
Tertiary care institution in Balochistan	24,037 (12.8)
Mode of presentation	
Ambulance	75,072 (39.9)
With respiratory support	20,825 (27.7)
With loss of consciousness	45,806 (61.0)
With active bleeding	2,092 (2.8)
Not specified	6,349 (8.5)
Nonambulance	113,231 (60.1)
Time of presentation	
Morning shift (8:00 am to 2:00 pm)	67,883 (36.0)
Evening shift (2:00 pm to 8:00 pm)	71,654 (38.1)
Night shift (8:00 pm to 8:00 am)	48,766 (25.9)
Day of presentation	
Weekdays (Monday to Friday)	91,211 (48.4)
Weekends (Saturday and Sunday)	97,092 (51.6)

As shown in Table 2, neonates accounted for the highest percentage of triage level 1 patients (36.6%), and children aged 1 to 5 years accounted for the highest percentage of triage levels 2 and 3 (38.5%) and triage levels 4 and 5 (55.7%) patients.

**Table 2.** Distribution of Patients by Triage Level (n=188,303)

	Triage Level 1	Triage Levels 2 and 3	Triage Levels 4 and 5
	n=22,388	n=128,131	n=37,784
Patient Age	Number of Patients (%)	Number of Patients (%)	Number of Patients (%)
0-1 month (neonates)	8,183 (36.6)	8,749 (6.8)	20 (0.1)
>1-12 months	6,247 (27.9)	39,871 (31.1)	12,353 (32.7)
>1-5 years	4,305 (19.2)	49,353 (38.5)	21,046 (55.7)
>5-10 years	2,679 (12.0)	27,069 (21.1)	1,018 (2.7)
>10 years	974 (4.4)	3,089 (2.4)	3,347 (8.9)

Table 3 shows the clinical diagnoses for 171,351 children in the cohort (all children excluding the neonates), and the Figure shows the organ systems involved. These diagnoses were provisional and principally clinical, supported by baseline laboratory investigations or imaging available in the ED. All diagnoses were either made by the on-call CMO or the on-call senior resident of the relevant subspecialty. The diagnoses of 8,936 children (5.2%) were either missing from the data or could not be made in the ED.

**Table 3.** Clinical Profile of Children Ages >1 Month to 14 Years Presenting to the National Institute of Child Health Emergency Department in 2017 (n=171,351)^a^

Organ System	Clinical/Provisional Diagnosis	Number of Patients (%)
Respiratory system		63,990 (37.3)
	Lower respiratory tract infection	30,649 (47.9)
	Upper respiratory tract infection	25,912 (40.5)
	Acute exacerbation of asthma/status asthmaticus	5,840 (9.1)
	Pleural effusion	506 (0.8)
	Stridor	509 (0.8)
	Croup	480 (0.8)
	Pneumothorax	94 (0.2)
Gastrointestinal system		28,130 (16.4)
	Acute gastroenteritis	25,881 (92)
	Appendicitis	1,147 (4.1)
	Acute liver failure	581 (2.1)
	Acute abdomen/peritonitis	324 (1.2)
	Constipation	181 (0.6)
	Liver abscess	16 (0.1)
Multisystem involvement		27,217 (15.9)
	Sepsis/shock	18,250 (67.1)
	Measles/mumps/chicken pox	3,872 (14.2)
	Malaria	2,818 (10.4)
	Poisoning/drug overdose	1,132 (4.2)
	Allergy/anaphylaxis	1,054 (3.9)
	Leukemia/lymphoma	91 (0.3)
Nervous system		19,356 (11.3)
	Febrile fit	9,742 (50.3)
	Meningitis/encephalitis	6,547 (33.8)
	Epilepsy/status epilepticus	1,758 (9.1)
	Cerebral malaria	876 (4.5)
	Space-occupying lesion	168 (0.9)
	Acute flaccid paralysis	154 (0.8)
	Intracranial bleed/stroke	111 (0.6)
Trauma		10,698 (6.2)
	Animal bite	7,176 (67.1)
	Road traffic accidents	3,118 (29.1)
	Burn	137 (1.3)
	Foreign body ingestion	94 (0.9)
	Tetanus	86 (0.8)
	Head injury	65 (0.6)
	Electric shock	12 (0.1)
	Gunshot	8 (0.1)
	Child abuse	2 (0.0)
Genitourinary system		5,255 (3.1)
	Urinary tract infection	3,196 (60.8)
	Renal colic	1,127 (21.4)
	Known/suspected nephrotic syndrome	565 (10.8)
	Suspected acute glomerulonephritis	151 (2.9)
	Acute kidney injury	126 (2.4)
	Hypertensive emergency/crisis	90 (1.7)
Hematologic system		2,873 (1.7)
	Nutritional anemia	1,528 (53.2)
	Known/suspected thalassemia	1,100 (38.3)
	Known/suspected aplastic anemia	61 (2.1)
	Febrile neutropenia	19 (0.7)
	Others	165 (5.7)
Cardiovascular system		2,309 (1.3)
	Septal defects	1,311 (56.8)
	Tet spell	280 (12.1)
	Patent ductus arteriosus	157 (6.8)
	Myocarditis	150 (6.5)
	Congestive cardiac failure	150 (6.5)
	Transposition of great arteries	135 (5.8)
	Arrhythmia	126 (5.5)
Endocrine/metabolic system		1,347 (0.8)
	Electrolyte imbalance	848 (63.0)
	Diabetic ketoacidosis	342 (25.4)
	Hypoglycemia	128 (9.5)
	Adrenal crisis	15 (1.1)
	Inborn errors of metabolism	14 (1.0)
Earache/infections		1,240 (0.7)
Missed diagnosis		8,936 (5.2)

^a^Diagnoses for neonates (age 0 to 1 month) are not included in this table.

**Figure.**
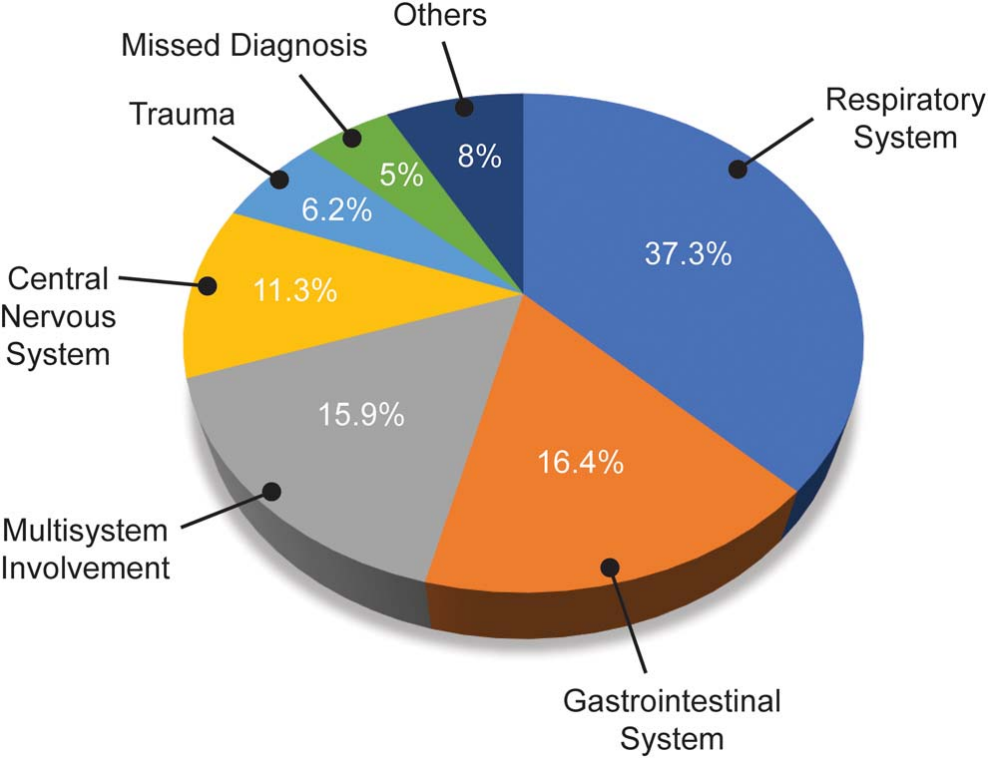
**Organ systems involved with the common illnesses among children age >1 month to 14 years presenting to the pediatric emergency department at the National Institute of Child Health in Karachi, Pakistan (n=171,351).**

Respiratory system diseases were the most common (37.3%), with upper and lower respiratory tract infections such as pharyngitis, tonsillitis, bronchitis, bronchiolitis, and pneumonia accounting for the majority of the illnesses in this category (88.4%). The second most common system involved was gastrointestinal (16.4%), with acute gastroenteritis accounting for most of the diagnoses (92.0%).

When children with upper and lower respiratory tract infections and acute gastroenteritis were compared for seasonal presentation, we found that respiratory tract infections were most common during the last quarter of the year (October to December), with 23,289 of 56,561 respiratory tract infection presentations (41.2%) during these winter months. Acute gastroenteritis presentations were most common during the second quarter of the year (April to June), with 10,008 of 25,881 cases (38.7%) registered during these hot summer months.

The clinical and provisional diagnoses of the neonates are shown in Table 4. Septic neonates accounted for the highest percentage of cases (23.8%). These cases may be early onset as a result of unsafe or contaminated delivery or late-onset neonatal sepsis. The next most common diagnoses were low birth weight or preterm babies requiring incubators and radiant warmers (18.4%), neonates with respiratory distress/pneumonia (15.2%), and neonates referred because of birth hypoxia/anoxia (13.2%).

**Table 4.** Clinical Profile of Children Ages 0-1 Month (Neonates) Presenting to the National Institute of Child Health Emergency Department in 2017 (n=16,952)

	Number of
Clinical/Provisional Diagnosis	Patients (%)
Sepsis	4,030 (23.8)
Low birth weight/preterm	3,112 (18.4)
Respiratory distress syndrome/pneumonia	2,578 (15.2)
Birth hypoxia/anoxia	2,231 (13.2)
Meconium aspiration syndrome	1,873 (11.0)
Neonatal jaundice	1,558 (9.2)
Transient tachypnea of newborn	613 (3.6)
Metabolic fits	373 (2.2)
Hemolytic disease of the newborn	122 (0.7)
Tracheoesophageal fistula	111 (0.7)
Acute abdomen	86 (0.5)
Cyanotic heart disease	82 (0.5)
Necrotizing enterocolitis	58 (0.3)
Suspected inborn errors of metabolism	62 (0.4)
Imperforate anus	45 (0.3)
Congenital diaphragmatic hernia	18 (0.1)

## DISCUSSION

Several essential and unknown facts emerged in our study regarding the spectrum of illnesses of children presenting to the largest pediatric ED in Karachi, Pakistan. The department managed a total of 188,303 children in 1 year, for an average of 515.9 patients per day. Sixty-eight percent of these patients were triage levels 2 and 3, and 11.9% were critically ill (triage level 1).

According to a retrospective study of the clinical profiles and outcomes of children who presented to the NICH ED in 2014, 172,162 patients were registered (an average of 471.7 patients per day), and 8% of these patients were triage level 1.^12^ Forty-eight percent of the triage level 1 patients were neonates. In comparison to these figures from 2014, our study found an overall increase in patient presentation of 9.3%. The proportion of triage level 1 patients also increased compared to 2014. However, compared to 2014, 2017 had a lower percentage of neonates in triage level 1 (48% vs 36.6%, respectively). We attribute this decrease to improved neonatal resuscitation and better management of sick babies at secondary care hospitals.

In the 2014 analysis, children aged 1 to 5 years accounted for 17% of the sample, infants for 22%, neonates for 48%, and older children (>5 years) for 13% of the sample.^12^ In the current study, children aged 1 to 5 years were most frequently seen in the ED (39.7%), followed by infants (31.1%). Older children (>5 years) accounted for 20% of the sample. Only 9% were neonates. The change in age at the time of presentation over these years likely reflects improved neonatal care as mentioned earlier and also indicates increasing illnesses among young children (infants up to 5 years).

Analysis of a nationwide Pakistani database (2010 to 2011) from general EDs showed injury to be the most frequent cause of ED visits (39%), followed by cases of gastrointestinal (18%), respiratory (14%), and neurologic (13%) etiologies.^11^ Almost half of the children (47%) were managed and discharged; 9% needed admission. The ED mortality rate was 1.3%. Of all the children visiting the ED, 65% were aged 10 to 16 years. A possible reason behind older children presenting to general EDs can be the misconception among parents that their children are now young adults and no longer categorized as children. However, easy accessibility of a hospital and earliest availability in an ED could also be factors in general ED presentations. Most of the centers included in the Atiq et al analysis are tertiary care and have central location.^11^ Most accident and imagery cases are referred to these hospitals, especially in cases of mass accidents, accounting for the high incidence of injuries reported in the study.

The illnesses accounting for the most visits to our ED included respiratory tract infections, acute gastroenteritis, sepsis, meningitis, seizures (epileptic and febrile fits), and trauma in infants and older children. In neonates, the ED burden was principally attributable to sepsis, low birth weight/preterm babies, respiratory distress, birth hypoxia/anoxia, meconium aspiration, and neonatal jaundice. The patterns of illnesses reported from other pediatric EDs are not much different.^7,10^ In a small study conducted in India, respiratory (29.8%), gastrointestinal/hepatic (16.3%), and neuroinfectious (15.6%) conditions were the common presenting illnesses.^16^ Only 1.7% of children were in triage level 1, 25% in level 2, 47.1% in level 3, and 26.1% in level 4. Mortality was highest among critically ill (triage level 1) patients (35.2%). In a larger study from Egypt, common presentations included respiratory distress and wheezy chest, followed by convulsions.^17^

Referral from the outpatient department of the NICH to the ED contributed to the morning peak hour observed in this study (11:00 am to 12:00 pm). Patients are referred for immediate administration of drugs, nebulization, or observation for deterioration of symptoms. Patients who were unsuccessfully managed at home or at secondary care facilitates during the night are also referred to our ED in the morning. The evening peak hours (6:00 pm to 8:00 pm) may also be attributed in part to the unavailability of affordable pediatricians in the evenings.

### Recommendations

In 2018, UNICEF declared Karachi as the most dangerous city in the world for a baby to be born.^18^ NICH is the only public pediatric hospital in Karachi. The hospital handles an undivided annual burden of almost 200,000 pediatric patients, principally from within Karachi but also from all other parts of Pakistan. Developing the ability of clinicians at secondary care centers to recognize and efficiently stabilize critical emergencies could potentially contribute to controlled patient infiltration in the large EDs. Training in basic life support and pediatric advanced life support is mandatory for ED doctors in tertiary care institutions. However, we recommend that this training be made mandatory for all nurses and doctors serving in secondary care pediatric EDs to enhance their ability to deal with the entire spectrum of pediatric emergencies. Special focus and refresher training should be scheduled for all presentations of respiratory, gastrointestinal, and neurologic emergencies that account for the majority of ED presentations. The nursing staff in most tertiary care pediatric EDs is well trained to accurately classify patients so that no critical child is missed and no unnecessary burden is placed on the ED by nonemergent presentations. Similar training should be made compulsory for nursing staff serving in secondary care pediatric EDs.

Additional studies from this institute and other pediatric institutes should investigate the trends of pediatric emergency care. The causes behind the high incidence of neonatal sepsis should be investigated. Access to vaccines for tetanus, influenza, and pneumococcal diseases should also be investigated.

### Limitations

Limitations of this study are that we did not consider symptoms at the time of presentation; did not segregate patients who primarily presented to the pediatric ED from those who were referred from a secondary care pediatric or general ED; and did not follow patients to assess their outcomes in terms of mortality, hospital admission, or discharge from the ED. Although we considered Saturday and Sunday to be weekend days in this study, some children attend school on Saturdays. The bias could not be removed. Similarly, during summer and winter breaks, the difference between weekdays and weekends has no value.

## CONCLUSION

This tertiary care pediatric emergency department at the NICH in Karachi, Pakistan has witnessed an increasing number of critical emergencies over time. The data show that respiratory and gastrointestinal emergencies account for the majority of the ED burden, with evenings and weekends being the busiest times. Support in stabilizing emergencies from secondary care EDs could potentially help streamline patient infiltration. Nationwide, longitudinal studies that enroll Pakistani children from both pediatric and general EDs and incorporate thorough data and technical data analysis strategies should be conducted. Such studies will contribute to understanding pediatric emergency trends and form the basis for developing nationwide recommendations to improve pediatric emergency care.

## Notes

This article meets the Accreditation Council for Graduate Medical Education and the American Board of Medical Specialties Maintenance of Certification competencies for Patient Care and Medical Knowledge.
